# Heritable and Precise Zebrafish Genome Editing Using a CRISPR-Cas System

**DOI:** 10.1371/journal.pone.0068708

**Published:** 2013-07-09

**Authors:** Woong Y. Hwang, Yanfang Fu, Deepak Reyon, Morgan L. Maeder, Prakriti Kaini, Jeffry D. Sander, J. Keith Joung, Randall T. Peterson, Jing-Ruey Joanna Yeh

**Affiliations:** 1 Cardiovascular Research Center, Massachusetts General Hospital, Charlestown, Massachusetts, United States of America; 2 Department of Medicine, Harvard Medical School, Boston, Massachusetts, United States of America; 3 Molecular Pathology Unit, Center for Cancer Research, and Center for Computational and Integrative Biology, Massachusetts General Hospital, Charlestown, Massachusetts, United States of America; 4 Department of Pathology, Harvard Medical School, Boston, Massachusetts, United States of America; 5 Broad Institute, Cambridge, Massachusetts, United States of America; Mayo Clinic, United States of America

## Abstract

We have previously reported a simple and customizable CRISPR (clustered regularly interspaced short palindromic repeats) RNA-guided Cas9 nuclease (RGN) system that can be used to efficiently and robustly introduce somatic indel mutations in endogenous zebrafish genes. Here we demonstrate that RGN-induced mutations are heritable, with efficiencies of germline transmission reaching as high as 100%. In addition, we extend the power of the RGN system by showing that these nucleases can be used with single-stranded oligodeoxynucleotides (ssODNs) to create precise intended sequence modifications, including single nucleotide substitutions. Finally, we describe and validate simple strategies that improve the targeting range of RGNs from 1 in every 128 basepairs (bps) of random DNA sequence to 1 in every 8 bps. Together, these advances expand the utility of the CRISPR-Cas system in the zebrafish beyond somatic indel formation to heritable and precise genome modifications.

## Introduction

Genome engineering represents a significant bottleneck for the utility of some of the most important biomedical model organisms, such as zebrafish. In particular, in order to faithfully generate human disease models, it is advantageous to develop a gene-editing technology that enables precise and predefined sequence modifications in the genome of a model organism.

The clustered regularly interspaced short palindromic repeats (CRISPR)/CRISPR-associated (Cas) systems are adaptive immune systems evolved in bacteria and archaea to defend against intruding viruses and plasmid DNAs [1]–[3]. Previously, Jinek et al. demonstrated that the Cas9 protein of a type II CRISPR/Cas system from *Streptococcus pyogenes* is a dual RNA-guided endonuclease [4]. Cas9 is directed by CRISPR RNA (crRNA), which confers target DNA recognition via sequence complementarity and base-pairing, and a trans-activating crRNA (tracrRNA) to mediate site-specific double-stranded DNA cleavage. Furthermore, these authors showed that *in vitro* Cas9 activity could be directed by a programmable, single chimeric RNA composed of both crRNA and tracrRNA sequences. Subsequently, various groups used the CRISPR-Cas system to perform targeted genome editing in cultured human cells, yeast and bacteria [5]–[10]. Using zebrafish embryos, we also demonstrated that a CRISPR RNA-guided nuclease (RGN) could be used effectively in whole organisms by introducing non-homologous end-joining (NHEJ)-mediated indel mutations in eight of ten endogenous genes we targeted [11].

**Figure 1:**
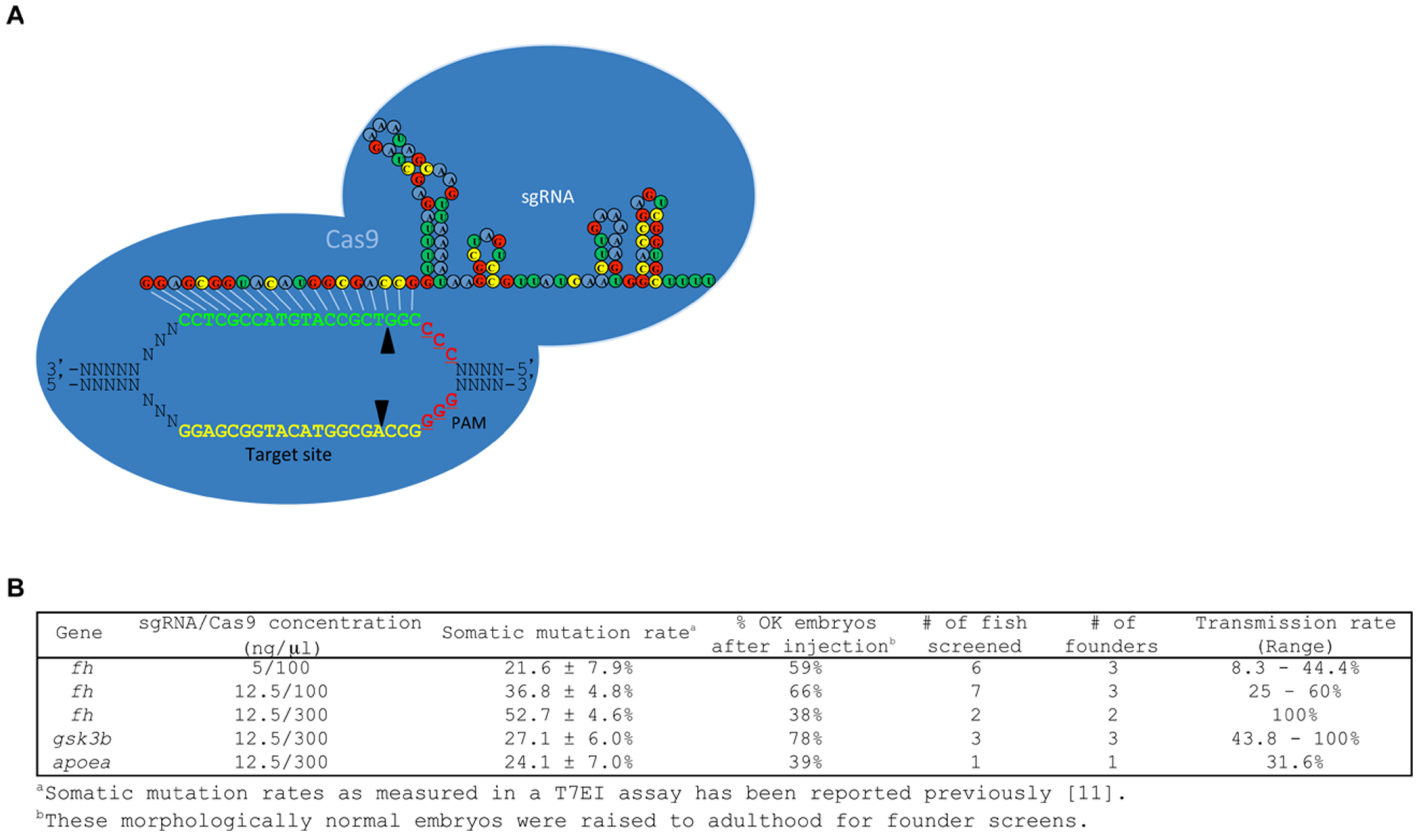
Engineered RGNs induces heritable gene disruption. (A) Schematic illustration of the RGN system. Engineered sgRNA:Cas9 system is depicted here based on the target sequence of the *fh* gene. sgRNA interacts with the complementary strand of the DNA target site harboring a 3′ protospacer adjacent motif (PAM) sequence (NGG) (yellow and red underlined text, respectively). sgRNA also interacts with Cas9 endonuclease (blue shape), resulting in DNA double-strand breaks (DSBs) at the target site. The reverse complement of the target site is highlighted as green text and the reverse complement of the PAM site is shown as red underlined text. The potential cleavage sites of Cas9 are indicated by arrowheads. This graphic representation is modified from a previous publication [11]. (B) Mutation frequencies in the germline induced by engineered RGNs. Fish that have been injected with sgRNA and Cas9 mRNA at the 1-cell stage were screened for founders. The concentrations of the sgRNA and the Cas9 mRNA are as indicated. The somatic mutation rates induced by these combinations have been reported previously [11]. The percentages of the injected embryos that developed normally at 1 day post-fertilization are shown. The sequences of the indel mutations identified in the germline are shown in Figure 2.

Our previous experiments utilized an RGN system consisting of a synthetic single guide RNA (sgRNA) that directs the monomeric Cas9 endonuclease from *S. Pyogenes* to induce site-specific DNA double-stranded breaks (DSBs) [11]. The first 20 nucleotides (nts) at the 5′ end of the ∼100-nt sgRNA are customized to complement a genomic target DNA adjacent to a 3′ NGG sequence, referred to as the protospacer adjacent motif (PAM), that is required for *S. Pyogenes* Cas9 activity (Figure 1A) [4]. Customized sgRNAs and Cas9-encoding mRNA were transcribed *in vitro* from a T7 promoter and co-injected into one-cell stage zebrafish embryos. Due to their relative ease of construction, RGNs are appealing alternatives to other tools such as TALENs and ZFNs for genome editing [12]–[14].

In the current study, we demonstrate several advances that will significantly broaden the applicability of the RGN system. First, we provide evidence that RGN-mediated mutations are heritably transmitted through the zebrafish germline. Second, we show that RGNs can be used with single-stranded oligodeoxynucleotides (ssODNs) to precisely introduce template-directed alterations including single-nucleotide mutations to zebrafish. Finally, we report on simple strategies that can be used to expand the targeting range of our sgRNA:Cas9 system from 1 in 128 bps of random sequence to 1 in 8 bps.

## Results

### High Germline Transmission Rates of RGN-induced Mutations

**Figure 2:**
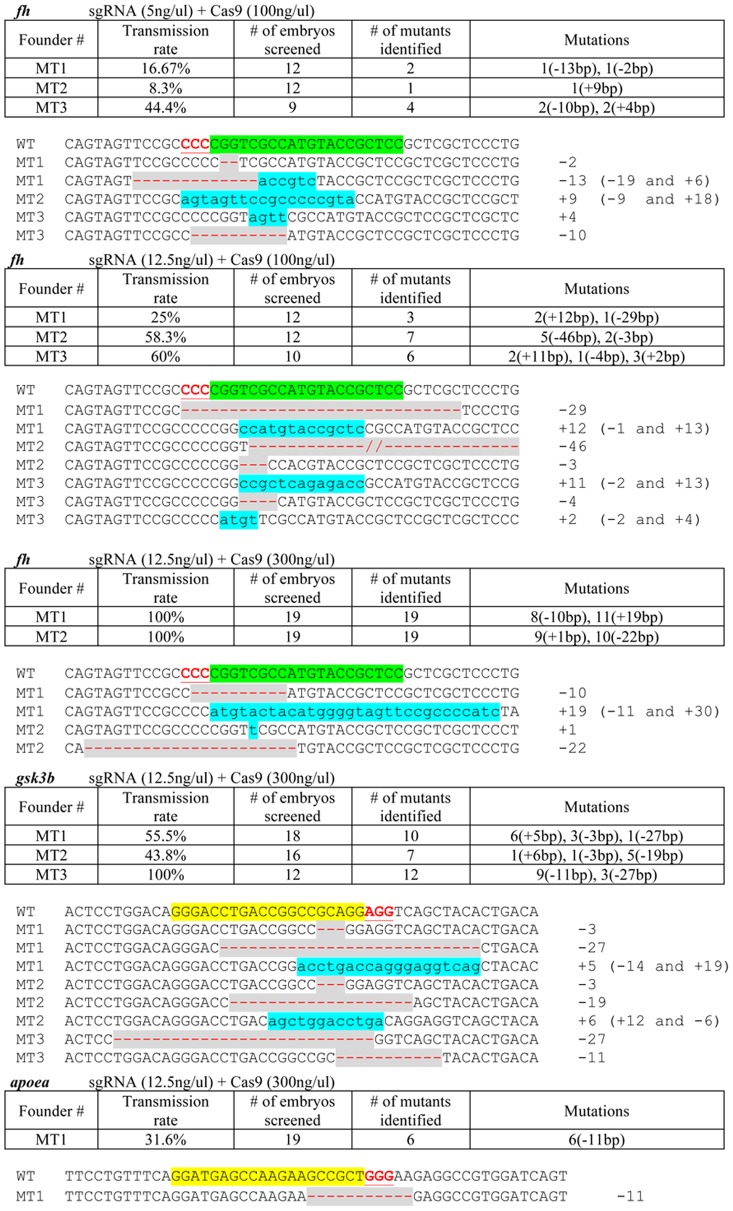
Germline mutation frequencies and sequences induced by engineered RGNs at the *fh*, *gsk3b* and *apoea* genes. Fish that had been previously injected with engineered RGNs were screened for founders carrying heritable mutations. The tables summarize the founder screening results. The concentrations of sgRNA and Cas9 mRNA used for microinjection are indicated on top of each table. In the last column of each table, the numbers of embryos that possess indel mutations are shown outside of the parentheses, and the sizes of the indels are shown inside the parentheses.+, insertion; −, deletion. The mutant sequences identified in the germline are shown beneath each table. The wild-type sequence is shown at the top with the target sites highlighted in yellow and the PAM sequence highlighted as red underlined text. For some genes, the target site is on the reverse complement strand and in these cases the reverse complement of the target site is highlighted in green and the reverse complement of the PAM site is highlighted as red underlined text. Deletions are shown as red dashes highlighted in grey and insertions as lower case letters highlighted in blue. The net change in length caused by each indel mutation is to the right of each sequence (+, insertion; –, deletion). Note that some alterations have both insertions and deletions of sequence and in these instances the alterations are enumerated in the parentheses.

To determine whether RGNs can induce heritable mutations, we screened fish that had been injected with sgRNAs targeted to three of the genes described in our initial report for their abilities to transmit RGN-mediated mutations to their progeny. As shown in Figures 1B and 2, we successfully identified founders that could pass RGN-induced mutations through the germline in all three genes. For the *fh* gene, we have previously reported the somatic mutation efficiencies induced by injections of various concentrations of sgRNA and Cas9 mRNA [11]. We found that injection of the solution containing 12.5 ng/µl of sgRNA and 300 ng/µl of Cas9 mRNA resulted in the highest somatic mutation rate, while it also induced the highest amount of toxicity as shown by the percentage of embryos that developed normally after injection (Figure 1B). Thus, we examined if the somatic mutation rates would have a positive correlation with the germline mutation rates, or if Cas9 at a higher concentration might pose a negative impact on germline transmission of the mutations. Here, we show that all three concentrations induce heritable mutations efficiently. In particular, for the 12.5 ng/µl of sgRNA and 300 ng/µl of Cas9 mRNA combination, we found that two out of two fish screened were founders and 100% of their offspring carried RGN-mediated indel mutations, suggesting that these two founders carry bialleleic mutations in the majority of their germ cells. In addition, we could not detect any wild-type *fh* allele in their fin biopsies by PCR, suggesting that these two fish also possess 100% or nearly 100% of bialleleic indel mutations in their tails (Figure S1).

In addition, from the fish previously injected with the RGN targeting *gsk3b*, we identified three founders out of three fish screened, one of which showed 100% germline transmission of the *gsk3b* mutations (Figures 1B and 2). Genotyping of the fins of the founders also indicated that the fish that showed 100% germline transmission had almost complete loss of the wild-type allele (Figure S2). For the *apoea* target site, we only screened one fish, and we found that six of the nineteen embryos that we tested carried an indel mutation (Figures 1B and 2). We have previously generated customized TALENs targeting these three genes - *fh*, *gsk3b* and *apoea* [11], [15]. The TALEN target sites either overlap with or are close to the RGN target site on the same gene (Figure S3). We found that except for the TALENs targeting *gsk3b*, which were ineffective, other RGNs and TALENs that target the same genes showed comparable somatic and germline mutation efficiencies (Figure S3). In addition, as has been observed in the founders produced by highly efficient TALEN pairs [15], founders produced by RGNs sometimes carry more than one type of mutation at their target loci (Figure 2). These results indicate that RGNs can efficiently induce heritable knockout mutations in zebrafish.

### Precise Targeted Insertion Using CRISPR-Cas and Single-stranded Oligo DNA

**Figure 3:**
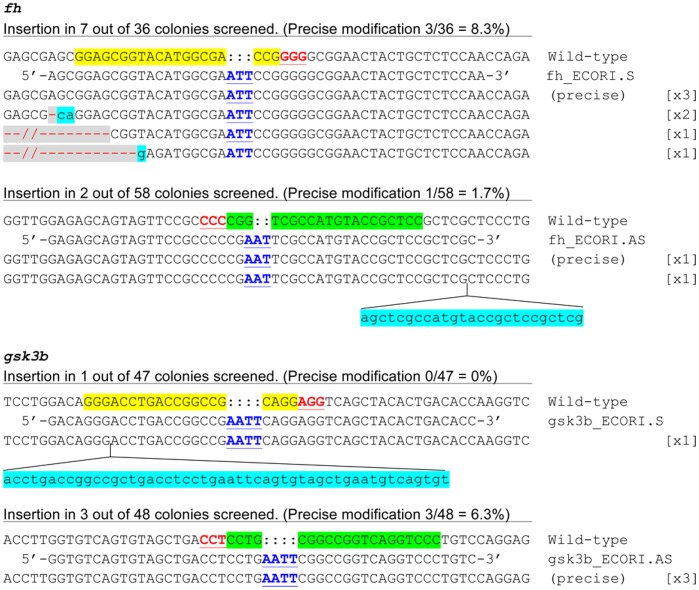
Targeted insertions achieved by co-injection of single-stranded oligonucleotides (ssODNs) and the RGN system. The sgRNAs targeting *fh* and *gsk3b* have been described previously [11]. For each gene, the wild-type sequence is shown at the top with the target site highlighted in yellow and the PAM sequence highlighted as red underlined text. For some cases the target sites are highlighted in green if the target sequences are in the reverse complement strand. The ssODNs containing 3–4 nucleotide (nt) insertions are shown beneath the wild-type sequences. The targeted insertions are highlighted as blue underlined capital letters. The target gene sequences identified in the injected embryos are shown beneath the ssODN sequences. Some of them contain only the precise intended changes (labeled as “precise” in parentheses on the right), while others contain additional indel mutations (deletions are shown as red dashes highlighted in grey and insertions as lower case letters highlighted in blue). The number of times each mutant sequence was isolated is shown in brackets.

To broaden the applications of RGNs in zebrafish, we investigated whether these nucleases might also facilitate precise targeted sequence modifications introduced by single-stranded oligodeoxynucleotides (ssODNs) in the injected animals. Previous work has shown that co-injection of TALENs and synthetic ssODNs can result in precise sequence modification of the zebrafish genome [16]. These ssODNs carried predefined mutations and ∼20 nts of flanking sequences homologous to the TALEN target loci. To test whether RGNs might also be used with ssODNs, we designed oligonucleotides containing sequences that either share sequence identity with the sgRNAs (denoted as “sense”) or are complementary to the sgRNAs (denoted as “anti-sense”) for two target sites. Each oligonucleotide also harbored a 3- or 4-bp insertion designed to create an *EcoR*I site (Figure 3). We co-injected these ssODNs with cognate sgRNA and Cas9 mRNA into one-cell stage zebrafish embryos. Subsequently, pools of ten injected embryos were used for amplification and subcloning of the sgRNA target loci. These colonies were screened by PCR and restriction digestion for the presence of the *EcoR*I site.

As shown in Figure 3, we found the intended precise sequence modifications in both genomic loci as verified by sequencing, although some of the modified alleles also contain additional indel mutations as previously described [16]. At the *fh* target site, the sense and antisense ssODN yielded 8.3% and 1.7% precise modification, respectively (among 36 and 58 PCR amplified sequences for the sense and antisense ssODN, respectively). At the *gsk3b* target site, the sense and antisense ssODN yielded 0% and 6.3% precise modification, respectively (among 47 and 48 PCR amplified sequences for the sense and antisense ssODN, respectively). We noticed that occasionally, a part of the sequences in the injected ssODN might be incorporated into the indels. The alleles that did not have the targeted modification at the intended position were not counted in our study. These data indicate that ssODNs can be used with RGNs to create precise targeted sequence modifications. In addition, these data suggest that there may be locus-dependent differences between the targeting rates of sense and antisense ssODNs. We examined the efficiencies of RGN-mediated DNA cleavage in the presence of the ssODNs and found that there are corresponding differences in the NHEJ-mediated mutation rates between those groups (Table S1), suggesting that some of the ssODNs may interfere with RGN activity.

### Precise Targeted Single-nucleotide Substitution by CRISPR-Cas

Next, we tested whether it might be possible to create a single bp mutation in the zebrafish genome using RGNs and ssODNs, something that has not previously been done with ZFNs or TALENs. To do this, we designed three ssODNs with a single nt substitution in either the protospacer adjacent motif (PAM) or the sgRNA target site 5 bp upstream of the PAM. We chose these locations because previous work has shown that the PAM sequence may be critical for cleavage and we wished to assess whether placement in or outside of the PAM might affect the efficiency with which we could recover the desired alterations.

**Figure 4:**
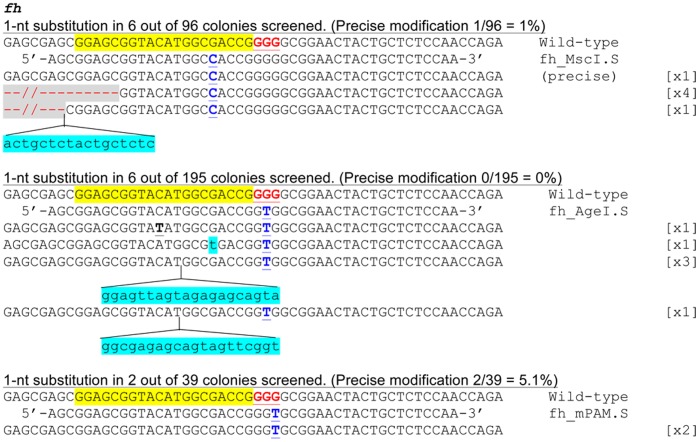
Single-nucleotide substitution achieved by co-injection of single-stranded oligonucleotides (ssODNs) and the RGN system. ssODNs carrying 1-nucleotide (nt) sequence substitutions (fh_MscI.S, fh_AgeI.S and fh_mPAM.S) were co-injected with Cas9 mRNA and the sgRNA targeting the *fh* gene. The wild-type *fh* sequence is shown at the top with the target site highlighted in yellow and the PAM sequence highlighted as red underlined text. The intended modifications are highlighted as blue underlined capital letters. The target gene sequences identified in the injected embryos are shown beneath the ssODN sequences. Some of them contain only the precise intended changes (labeled as “precise” in parentheses on the right), while others contain additional indel mutations (deletions are shown as red dashes highlighted in grey and insertions as lower case letters highlighted in blue). One of the identified sequence has a 1-bp point mutation (highlighted in bold and by an underline) in addition to the intended sequence. The number of times each mutant sequence was isolated is shown in brackets.

As shown in Figure 4, we successfully identified alleles carrying the desired 1-bp substitution at the target locus using all three ssODNs, although most of them also contained additional indel mutations. From a pool of embryos co-injected with the *fh* sgRNA, the Cas9 mRNA and the ssODN carrying a 1-nt substitution in the *fh* sgRNA target site, we recovered six out of 96 PCR amplified sequences that contained the intended 1-nt substitution, one of which had the precise sequence modification (without any unintended indels in neighboring sequence). Previously, Jiang *et al* have suggested that gRNA:Cas9 may cleave DNA sequences followed by either NGG or NNGG [8]. Since the *fh* target site is followed by 3′GGGG, we synthesized two ssODNs that carry a 1-nt substitution in the PAM sequence. With the first ssODN in which the PAM sequence was changed to GTGG, we recovered six out of 195 PCR amplified sequences containing the intended 1-nt substitution, but all six also contained other unintended sequence modifications in surrounding sequence. Nevertheless, with the second ssODN in which the PAM sequence was changed to GGTG, we recovered two out of 39 PCR amplified sequences (5.1%) that had the precise 1-nt substitution without any additional mutation. As in the targeted insertion experiments, we detected one allele that contained a part of the ssODN sequence in the indel without the targeted modification at the intended position (data not shown). This allele was not counted in our results. In sum, these results suggest that ssODNs and RGNs can be used in concert to induce single bp substitutions *in vivo*.

### Increasing RGN Targeting Range by Relaxing the sgRNA 5′ GG Requirement

**Figure 5:**
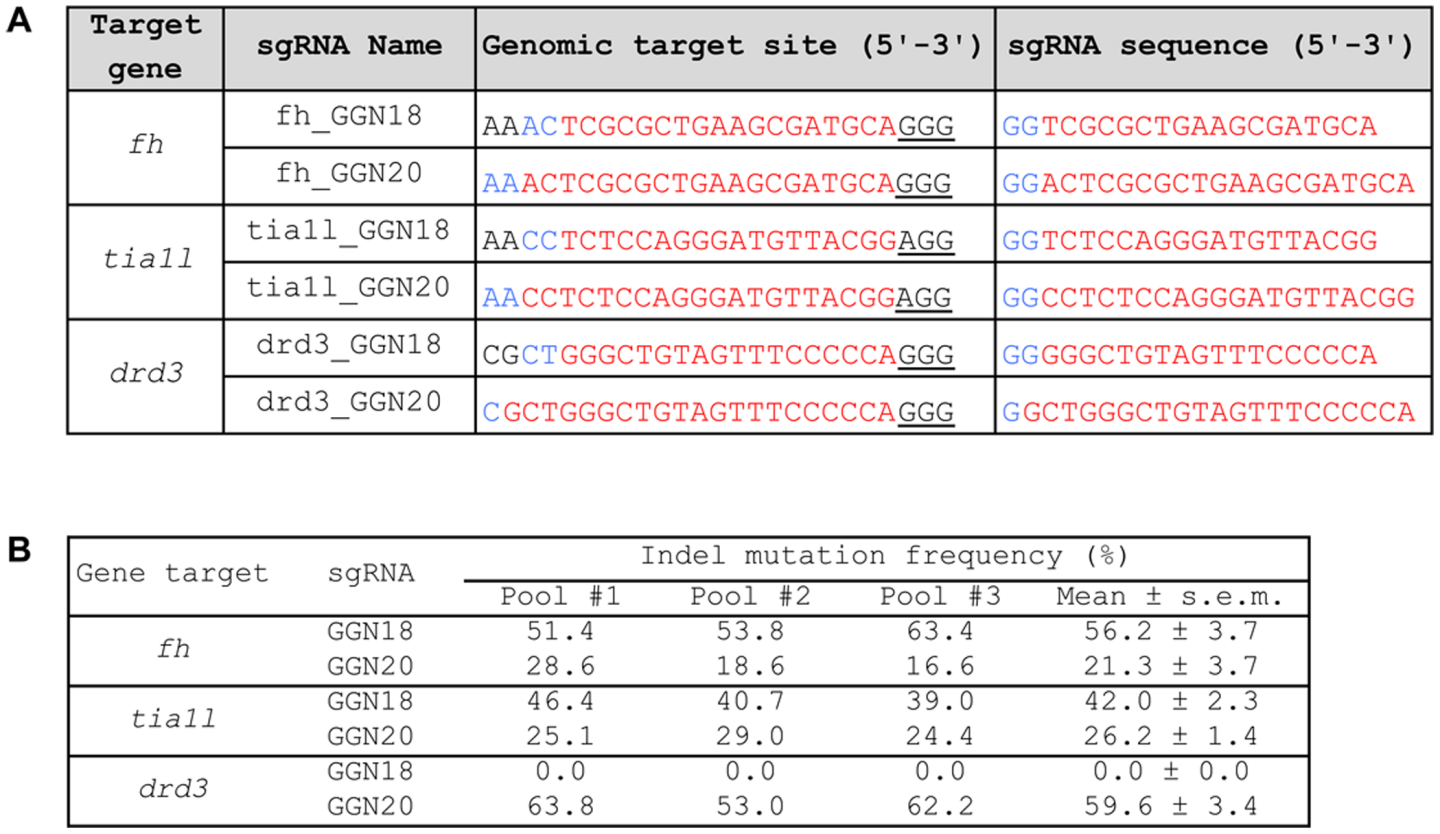
Somatic mutation frequencies induced by RGNs. (A) GGN18 and GGN20 sgRNAs and their genomic target sequences. Sequences of the variable regions of the sgRNAs are shown here. These sgRNAs contain 1–2 nt mismatches to their genomic target sequences at the 5′ end. sgRNAs bind to the reverse complement strand of the DNA that possess the genomic target sequences (see illustration in Figure 1A). Matching genomic and sgRNA sequences are marked in red, while the mismatches are marked in blue. PAM is underlined. (B) The indel mutation frequencies were assess using the T7EI assay.

Our current platform for sgRNA production relies on T7 RNA polymerase-mediated *in vitro* transcription. As a result, all of the sgRNAs produced using this platform must contain a GG dinucleotide at the 5′ end. This requirement together with the requirement for the NGG PAM sequence restricts the targeting range of RGNs to 1 in 128 bps of random DNA sequence. To attempt to improve the targeting range of our RGN platform, we explored whether sgRNAs could tolerate 2-nt mismatches at their 5′ ends. We chose three genes – *fh*, *tia1l* and *drd3*, and generated two sgRNAs per gene such that each sgRNA contained either 18 or 20 nts complementary to the genomic DNA target (denoted as GGN18 and GGN20, respectively). These sgRNAs also had additional GG at their 5′ ends that were not complementary to the genomic DNA targets (except for *drd3* GGN20, which had only 1-bp mismatch) (Figure 5A). As shown in Figure 5B, we found that all but one of the sgRNAs could efficiently induce site-specific mutations as judged by a T7 endonuclease I (T7EI) assay. We do not know exactly why the *drd3* GGN18 sgRNA failed to induce mutations. One potential explanation is that it might self anneal between five consecutive guanines and five consecutive cytosines in the customized region of this sgRNA (Figure 5A).

These results demonstrate that there are two potential strategies for broadening the targeting range of RGNs while still satisfying the T7 promoter requirement for a 5′ GG; either mismatching the two 5′ nucleotides of the gRNA (as in the GGN18 sgRNAs) or adding two additional Gs 5′ to the gRNA (as in the GGN20 sgRNAs). Thus, in theory, our RGN system can target any genomic DNA sequence followed by 3′NGG, which can be on either strand of the DNA. In conclusion, by relaxing the 5′GG requirement for the target sequence, the targeting range of the RGN system is increased to 1 in every 8 bps of random DNA sequence (see Materials and Methods).

## Discussion

We have previously described an engineered RGN system that can induce DSBs and NHEJ-mediated mutations in zebrafish embryos with high efficiency. Compared to other genome editing platforms, including TALENs and ZFNs, the RGN system is much simpler to implement. In this report, we provide confirmatory evidence that RGN-induced mutations can be passed through the zebrafish germline efficiently. In some cases, we found that 100% of the offspring from the founder fish possessed indel mutations at the target locus. Genotyping of the fins also suggests that these fish may have complete loss of the wild-type allele at the target locus. Thus, in at least two target loci, *fh* and *gsk3b*, we found that RGNs induced abundant biallelic mutations in the injected fish. Due to the high efficiency of the RGN system, in order to generate a zebrafish mutant line, researchers may wish to reduce the concentrations of the sgRNA and Cas9 mRNA if biallelic deletion of the gene of interest will result in a lethal phenotype. These results indicate that engineered RGNs are robust and easy-to-use tools for generating zebrafish knockouts and open the door to exciting potential applications for the RGN system, such as simultaneous disruption of multiple genes or induction of chromosomal deletions by multiplexing various sgRNAs for microinjection.

In addition, we explored whether RGNs might facilitate the introduction of precise genome modifications. The capability to introduce such modifications in zebrafish is desirable because it may facilitate the generation of human disease models, enable functional analysis of different protein domains, and render temporal or spatial control of gene deletion. Previously, two strategies have been used for this purpose – one approach uses double-stranded plasmid DNAs whereas the other uses ssODNs as the donor DNA in conjunction with TALENs [16], [17]. Although it remains to be seen whether RGNs may be used to replace TALENs in the former strategy, we find that RGNs can be used in concert with ssODNs to create precise and predefined insertions. We achieved 8.3% and 6.3% precise modifications for a targeted 3–4 bp insertion at the *fh* and *gsk3b* RGN target sites, respectively. During the preparation of this manuscript, Chang *et al* reported a case of imprecise genomic sequence modification while inserting mloxP sequence into a target locus using gRNA/Cas9 and a single-stranded DNA in zebrafish embryos [18]. In our study, we were able to achieve precise genomic sequence modification, without any unintended indels in flanking sequence. In addition, we noticed that co-injection of ssODNs sometimes interfered with the activities of RGNs irrespective of which strand of the sgRNA genomic target site the ssODNs are homologous to. Thus, the results suggest that it may be useful to test various ssODN sequences and optimize the efficiency when using this approach.

We show that the use of RGNs and ssODNs in concert can also facilitate precise single bp substitution in a zebrafish gene, which to our knowledge is the first demonstration of an approach for generating targeted point mutations in zebrafish. In this study, we made a 1-nt substitution at various positions in the RGN target locus. We attempted to change the nucleotide at the +5 position (the fifth nucleotide counting 3′ to 5′ from the PAM) and two of the nucleotides in the PAM. We observed 3.1–6.3% of PCR amplified sequences being modified at the targeted nucleotide using these three ssODNs (39 to 195 total sequences analyzed for each ssODN). However, when the modification is at the+5 position, only 16% of the sequences with the intended 1-nt substitution are precise (without any unintended mutations). None of the sequences with the targeted modification is precise when the PAM sequence is changed from GGGG to GTGG. Finally, both sequences with the targeted modification that we recovered are precise when the PAM sequence is changed from GGGG to GGTG. It will be interesting to see whether the differences in the rates of precisely modified alleles that we observed while targeting different positions of the *fh* RGN binding site can also be observed at other RGN target sites.

The somatic mutation rates that we observed for the targeted insertions and single bp substitutions are within the range of mutation efficiencies for which we and others have successfully identified founders using ZFNs and TALENs [15], [19]–[21]. Based on our past experience in multiple genes, at somatic mutation rates between 4.5 to 8%, we have successfully isolated one or more founders by screening 22 or less fish ([15], [19], [21] and unpublished results). In addition, at somatic mutation rates between 1 to 3%, we and others have also been able to isolate founders by screening more fish ([20], unpublished results and personal communications). Judging from the high rates of heritable indel mutations mediated by the RGN system, we expect that the ssODN-mediated modifications should be successful transmitted through germline.

Finally, we have provided two strategies for increasing the targeting range of our previously reported RGN system to 1 in 8 bps of random DNA by demonstrating that the requirement for specificity of the first two nucleotides at the 5′ end of sgRNAs is low. Clearly, more work will be needed to determine what other positions in the engineered RGNs are or are not important for specificity. These two strategies will significantly broaden the range of the DNA sequences that researchers will be able to target using this simple and easy-to-use method.

ZFNs, TALENs and the CRISPR/Cas system are three genome engineering platforms that have been successfully exploited in zebrafish [11], [15]–[17], [19], [20], [22]–[27]. In addition, many of the reagents and protocols for generating these customized DNA nucleases have been made publicly available [11], [16], [19], [21], [22], [25], [26], [28]–[34]. Among these three platforms, specific and efficient ZFNs are more challenging to engineer due to the context-dependent activities of zinc finger motifs. On the other hand, both TALENs and customized CRISPR/Cas can typically be designed with the primary target sequence alone, and they have both shown high efficiencies in general. Compared to the CRISPR/Cas system, TALENs are more costly to generate, but they have a better theoretical targeting range (at least three TALENs can be made for every single bp in random DNA sequence) [12]. Interestingly, we have observed four cases where a gene was efficiently mutated by either TALENs or CRISPR/Cas but not both, despite the fact that the two approaches targeted overlapping or nearby sequences [11]. Thus, it remains to be determined what additional factors may affect the functions of these two systems. For example, it has been shown that TALENs are sensitive to DNA methylation [35].

At present, there has not been a systematic or genome-wide study of the *in vivo* off-target effects of TALENs or CRISPR/Cas. However, off-target cleavage by TALENs has been reported in several individual studies. For example, in one study, TALENs discriminated the DNA sequence containing 6 bps but not 2 bps of mismatch to their intended 36-bp binding sequence in the injected zebrafish embryos [22]. In another study, off-target cleavage at a site with 2 bps of mismatch has also been detected, but at 20–30 fold reduced rates compared to that at the intended target site [15]. For RGNs, previous studies of the double RNA-guided Cas9 system have suggested that the last 12–14 nts (called the “seed” sequence) in the 20-nt variable region of the crRNA are most critical for directing Cas9 activity [4], [6]. However, it seems likely that different sgRNAs will have different degrees of specificity and each may have its own unique profile of off-target activities. Thus, more studies are needed in order to understand the extent of RGN off-target effects. No matter which genome engineering platform one chooses, it is always important to outcross the mutant lines multiple generations to eliminate any unlinked mutation, conduct rescue experiments whenever possible and validate the phenotypes with at least two independent lines.

In conclusion, this study demonstrates that engineered RGNs can be used in a wide range of applications for precise and heritable genome editing in zebrafish. The techniques described herein may also be extended for use in other organisms.

## Materials and Methods

### Zebrafish Care

All zebrafish care and uses were approved by the Massachusetts General Hospital Subcommittee on Research Animal Care.

### Cas9 Nuclease and Single Guide RNA (sgRNA) Expression Vectors

The Cas9 nuclease expression vector pMLM3613 used for *in vitro* transcription of the Cas9 mRNA has been described previously [11]. To construct a customized sgRNA expression vector, pDR274 harboring a T7 promoter positioned upstream of a partial guide RNA sequence was digested with BsaI [11]. A pair of oligonucleotides containing the sgRNA target sequence were annealed and cloned into this vector backbone. The annealed oligonucleotides have overhangs that are compatible with directional cloning into the BsaI-digested pDR274 vector. The genomic target sites and sequences of the oligonucleotides constructed in this study are listed in Table S2. In addition, plasmids pDR279, pDR299 and pDR338 encode sgRNAs that target sequences in the *fh*, *apoea* and *gsk3b* genes, respectively, and have been described previously [11]. pMLM3613, pDR274, pDR279, pDR299 and pDR338 are all available from Addgene (http://www.addgene.org/crispr-cas). Other constructs used in this study will be provided upon request.

### Production of sgRNA and Cas9 mRNA

sgRNAs were transcribed using DraI-digested sgRNA expression vectors as templates and the MAXIscript T7 kit (Life Technologies). Cas9 mRNA was transcribed using PmeI-digested Cas9 expression vector and the mMESSAGE mMACHINE T7 ULTRA kit (Life Technologies). Following completion of transcription, poly(A) tailing reaction and DNase I treatment were performed according to the manufacturer’s instructions for the Cas9-encoding mRNA. Both the sgRNA and the Cas9-encoding mRNA were then purified by either LiCl or ammonium acetate precipitation and re-dissolved in RNase-free water.

### Microinjection of Zebrafish Embryos

sgRNA and Cas9 mRNA were co-injected into one-cell stage zebrafish embryos. Each embryo was injected with approximately 2 nl of solution containing 12.5 ng/µl of sgRNA and 300 ng/µl of Cas9 mRNA unless otherwise indicated. If sgRNA:Cas9 mRNAs were co-injected with single-stranded oligonucleotide (ssODN), a solution containing 12.5 ng/µl of sgRNA, 200 ng/µl of Cas9 mRNA and 25–50 ng/µl of ssODN was used for microinjection. On the next day, injected embryos were inspected under stereoscope. Only embryos that developed normally were used for analyses. Genomic DNA was extracted from embryos at 1–2 days after injection for either T7 Endonuclease I assays or DNA sequencing experiments as described below.

### T7 Endonuclease I (T7EI) Mutation Detection Assay

Genomic DNA was extracted from pools of 3 control or injected embryos. Targeted genomic loci were amplified using primers designed to anneal approximately 150 to 200 base pairs upstream and downstream from the expected cut site and Phusion Hot Start II high-fidelity DNA polymerase (New England Biolabs) according to the manufacturer’s instructions. All of the primers used in this study have been described previously [11]. PCR products were purified with Ampure XP (Agencourt) according to the manufacturer’s instructions. T7 Endonuclease I assays were performed and estimated NHEJ frequencies were calculated as previously described [30].

### Identification of Indel and Targeted Mutations

Each target locus was amplified by PCR from pooled genomic DNA of ten injected embryos. The resulting PCR products were cloned into the pGEM-T vector (Promega) and transformed into bacteria. Thus, each colony represented one PCR amplified sequence. To determine the somatic mutation rates, plasmid DNAs isolated from single colonies were sequenced by the Massachusetts General Hospital DNA Sequencing Core. NHEJ-mediated indel mutation rates were determined by the numbers of colonies containing mutant sequences divided by the total numbers of colonies sequenced. To identify the targeted sequence modifications introduced by ssODNs, single colonies were lyzed in 50 µl of water and used for PCR. The resulting PCR products were screened for the presence of restriction sites indicative of targeted sequence modifications. For all of the ssODNs used in this study, the targeted mutations should yield new restriction enzyme sites (as indicated in the names of the ssODNs) not present in the wild-type sequences. Colonies possessing the targeted modifications as judged by the restriction digestion were then sequence verified.

### Founder Screen

Potential founders were crossed with wild-type zebrafish. Three to four days post-fertilization, progeny were lysed individually in 25 microliters of the alkaline lysis buffer (25 mM NaOH, 0.2 mM EDTA) and heated at 95°C for 30 minutes. Subsequently, the DNA solution was neutralized using 25 microliter of the neutralization buffer (40 mM Tris-HCl). Samples were spun at 3,000 rpm for 5 minutes, and the supernatant contained extracted genomic DNA. In general, 12 embryos from each potential founder were screened for the presence of indel mutations by PCR amplifying the region surrounding the relevant RGN cleavage site using a 6-FAM labeled fluorescent forward primer and a regular reverse primer. The fluorescent PCR products were analyzed on ABI 3730×l DNA analyzer to evaluate their sizes [29]. For sequence confirmation, genomic DNAs from single embryos were amplified using targeted loci-specific primers. The PCR products were treated with ExoSAP-IT (Affymetrix) according to the manufacturer’s instructions before submitting for Sanger sequencing. All of the primers used in this study have been described previously [11]. Mutant fish lines generated will be provided upon request.

### Targeting Range Calculation

Theoretically, the RGN system can target any genomic DNA sequence followed by 3′NGG, and the target site may be found on either strand of the DNA. Thus, the targeting range is ¼×¼×2 = 1/8, which means 1 in every 8 bps of random DNA sequence.

## Supporting Information

Figure S1 **Two** ***fh*** **founder fish showed a complete loss of the wild-type** ***fh*** **allele in their fins.**
(PDF)

Figure S2 **One** ***gsk3b*** **founder fish showed an almost complete loss of the wild-type allele in its fins.**
(PDF)

Figure S3 **TALENs and RGNs for the** ***fh*** **,** ***gsk3b*** **and** ***apoea*** **genes and their somatic and germline mutation efficiencies.**
(PDF)

Table S1 **Mutation frequencies in the embryos co-injected with ssODN and the sgRNA:Cas9 system.**
(PDF)

Table S2 **Sequences of the oligonucleotides used for constructing sgRNA constructs in this study.**
(PDF)
