# Supplementary material for: Heritable and Precise Zebrafish Genome Editing Using a CRISPR-Cas System
Source: PLoS One. 2013 Jul 9;8(7):e68708. doi: 10.1371/journal.pone.0068708 (PMC3706373; doi:10.1371/journal.pone.0068708)
Supplement: Figure S1 — Two fh founder fish showed a complete loss of the wild-type fh allele in their fins. (PDF) [file pone.0068708.s001.pdf]

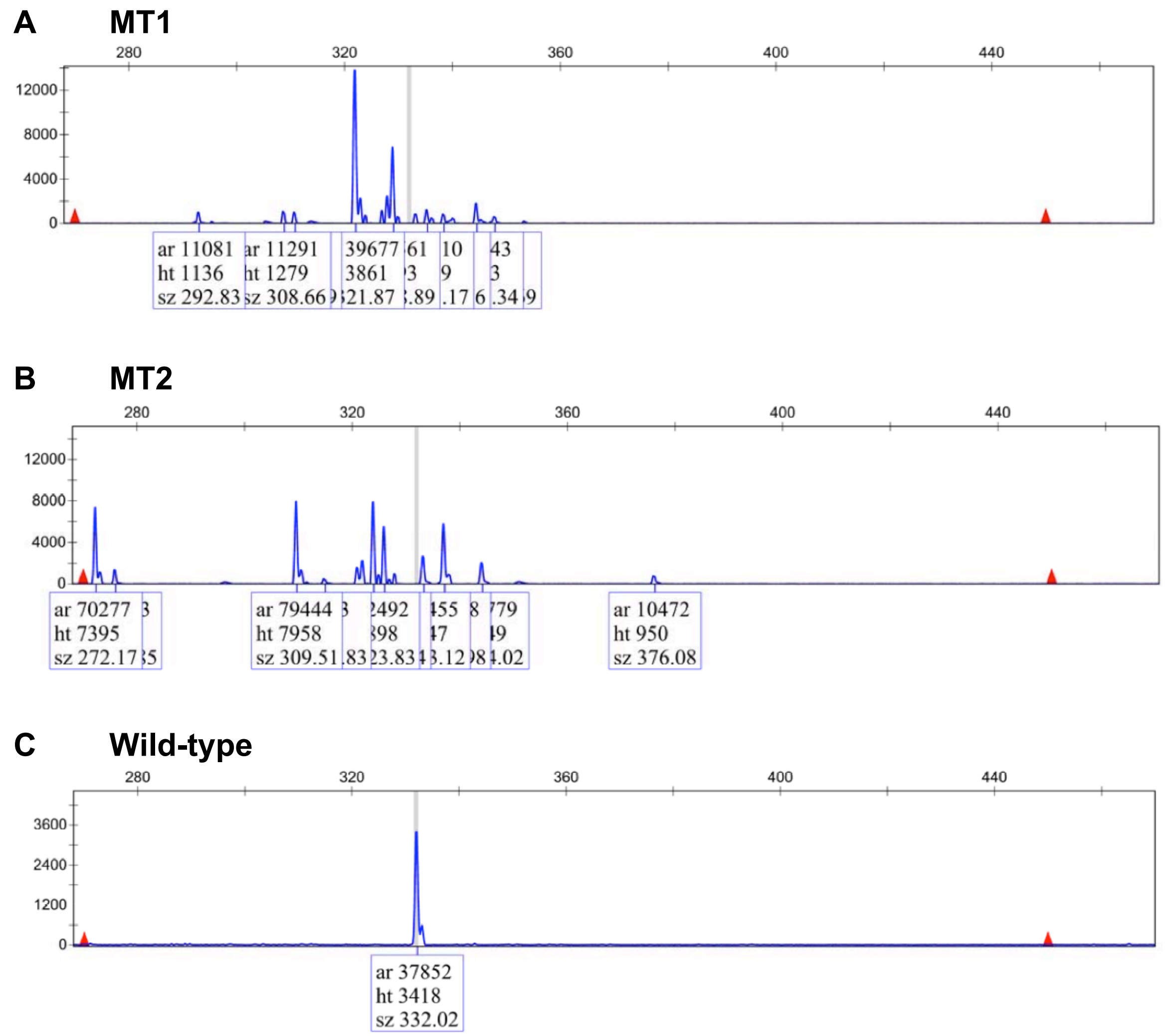

Figure S1. Two *fh* founder fish showed a complete loss of the wild-type *fh* allele in their fins. Genomic DNAs isolated from the fin biopsies of two *fh* founder fish (MT1 and MT2) obtained by injecting 12.5ng/ul of sgRNA and 300ng/ul of Cas9 mRNA were used for PCR amplification of the *fh* target locus. Fluorescent 6-FAM-labelled PCR products were analyzed using a DNA analyzer for size determination. (A) MT1. (B) MT2. (C) Wild-type. While the wild-type genomic DNA show only one PCR product of 332 bps, the PCR products in A-B are of various sizes indicating indel mutations in these founder fish.
