# Supplementary material for: Heritable and Precise Zebrafish Genome Editing Using a CRISPR-Cas System
Source: PLoS One. 2013 Jul 9;8(7):e68708. doi: 10.1371/journal.pone.0068708 (PMC3706373; doi:10.1371/journal.pone.0068708)
Supplement: Figure S2 — One gsk3b founder fish showed an almost complete loss of the wild-type allele in its fins. (PDF) [file pone.0068708.s002.pdf]

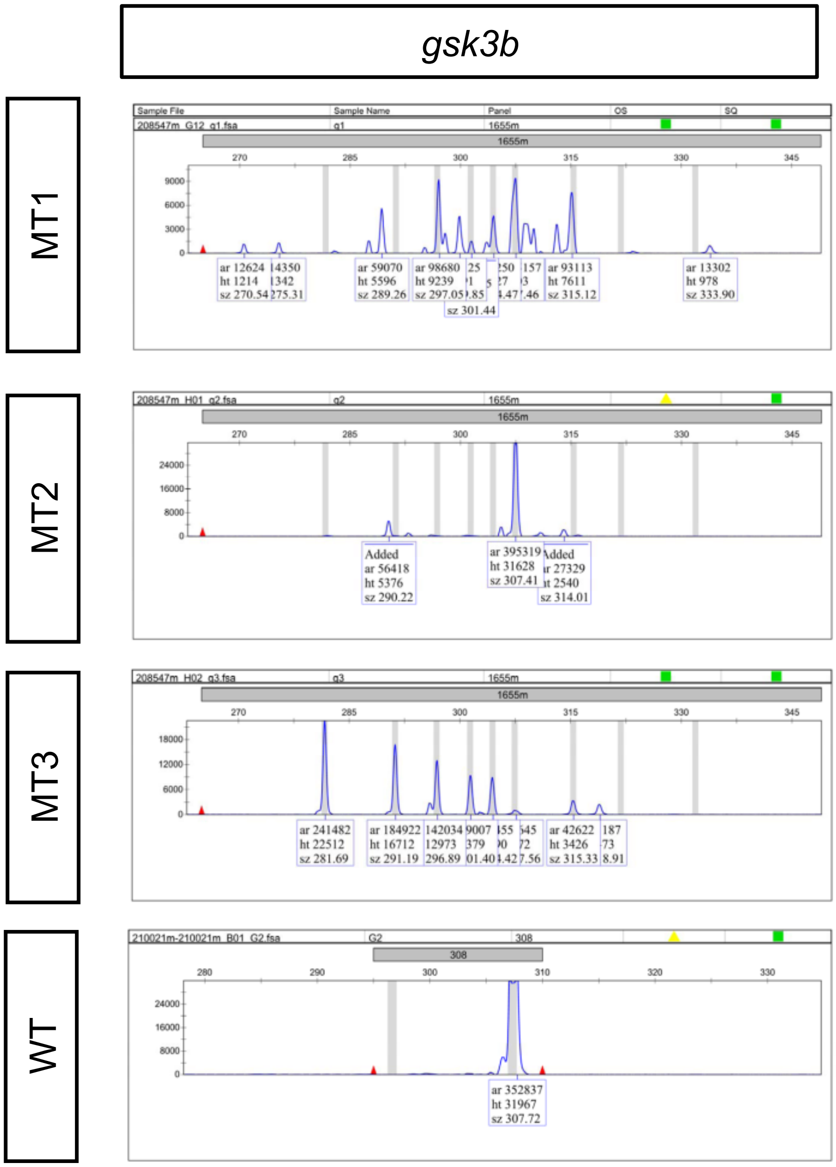

Figure S2. One *gsk3b* founder fish showed an almost complete loss of the wild-type allele in its fins. Genomic DNAs isolated from the fin biopsies of three *gsk3b* founder fish (MT1, MT2 and MT3) and a wild-type fish (WT) were used for PCR amplification of the *gsk3b* target locus. Fluorescent 6-FAM-labelled PCR products were analyzed using a DNA analyzer for size determination. While the wild-type genomic DNA show only one PCR product of 307 bps, the PCR products in the founders are of various sizes indicating indel mutations in these founder fish.
