# Supplementary material for: Heritable and Precise Zebrafish Genome Editing Using a CRISPR-Cas System
Source: PLoS One. 2013 Jul 9;8(7):e68708. doi: 10.1371/journal.pone.0068708 (PMC3706373; doi:10.1371/journal.pone.0068708)
Supplement: Figure S3 — TALENs and RGNs for the fh , gsk3b and apoea genes and their somatic and germline mutation efficiencies. (PDF) [file pone.0068708.s003.pdf]

A

fh
GCCCCCGGTCGCCATGTACCGCTCCGCTCGCTCCCTGCATCGCTTCAGCGCGAGTTTGTCAGATCTGCGGGCCGCTCAGAGATCCATCAAAGCTCG

gsk3b
CCACAGTGGTGGCGACTCCTGGACAGGGACCTGACCGGCCGCAGGAGGTCAGCTACACTGACACCAAGGTCATCGG

apoea
GCTGCCAGGGGCGATTCCTGTTTCAGGATGAGCCAAGAAGCCGCTGGGAAGAGGCCGTGGATCAGTTCTGGAACCAT

B

Gene	Nuclease[a]	Somatic mutation rate[b]	# of founder identified / # of fish screened	Germline transmission rate
fh	TALENs	60.0%	3/3	33.3 - 41.7%
fh	RGN	52.7%	2/2	100%
gsk3b	TALENs	0.0%		
gsk3b	RGNs	27.1%	3/3	43.8 - 100%
apoea	TALENs	20.6%	2/2	8.3 - 58.3%
apoea	RGNs	24.1%	1/1	31.6%

[a]TALENs were injected at 300 ng/ul; RGNs were injected at the dose combination of 12.5 ng/ul sgRNA:300 ng/ul of Cas9 mRNA.
[b]For TALENs, mutation rates were determined by colony sequencing as described in [1]. For RGNs, mutation rates were determined by T7EI assays [2].

Figure S3. TALENs and RGNs for the *fh*, *gsk3b* and *apoea* genes and their somatic and germline mutation efficiencies. (A) The genomic target sequences of TALENs and RGNs in *fh*, *gsk3b* and *apoea*. TALEN target sites are underlined, while the RGN target sites are highlighted in yellow or in green if the target site is on the reverse complement strand. (B) Comparison of the somatic and germline mutation rates of TALENs and RGNs at these three genes. Except for the founder screen data of the RGNs and the TALENs targeting *apoea*, the rest of the data have been reported previously [1,2].

References

1. Cade L, Reyon D, Hwang WY, Tsai SQ, Patel S, et al. (2012) Highly efficient generation of heritable zebrafish gene mutations using homo- and heterodimeric TALENs. Nucleic Acids Res 40: 8001-8010.
2. Hwang WY, Fu Y, Reyon D, Maeder ML, Tsai SQ, et al. (2013) Efficient genome editing in zebrafish using a CRISPR-Cas system. Nat Biotechnol 31: 227-229.
