# Supplementary material for: Heritable and Precise Zebrafish Genome Editing Using a CRISPR-Cas System
Source: PLoS One. 2013 Jul 9;8(7):e68708. doi: 10.1371/journal.pone.0068708 (PMC3706373; doi:10.1371/journal.pone.0068708)
Supplement: Table S1 — Mutation frequencies in the embryos co-injected with ssODN and the sgRNA:Cas9 system. (PDF) [file pone.0068708.s004.pdf]

Table S1. Mutation frequencies in the embryos co-injected with ssODN and the sgRNA:Cas9 system. Indel mutation frequencies shown here were assessed by colony sequencing as described in the Materials and Methods. n, the numbers of colonies sequenced.

sgRNA/Cas9 target gene	ssODN	
	EcoRI.S	EcoRI.AS
fh	81% (n=21)	28.5% (n=21)
gsk3b	28.1% (n=32)	70.4% (n=27)
