# Supplementary material for: Heritable and Precise Zebrafish Genome Editing Using a CRISPR-Cas System
Source: PLoS One. 2013 Jul 9;8(7):e68708. doi: 10.1371/journal.pone.0068708 (PMC3706373; doi:10.1371/journal.pone.0068708)
Supplement: Table S2 — Sequences of the oligonucleotides used for constructing sgRNA constructs in this study. (PDF) [file pone.0068708.s005.pdf]

Table S2. Sequences of the oligonucleotides used for constructing sgRNA constructs in this study.

sgRNA Name	Oligonucleotide 1 (5'-3')	Oligonucleotide 2 (5'-3')
fh_GGN18	TAGGTCGCGCTGAAGCGATGCA	AAACTGCATCGCTTCAGCGCGA
fh_GGN20	TAGGACTCGCGCTGAAGCGATGCA	AAACTGCATCGCTTCAGCGCGAGT
tia1l_GGN18	TAGGTCTCCAGGGATGTTACGG	AAACCCGTAACATCCCTGGAGA
tia1l_GGN20	TAGGCCTCTCCAGGGATGTTACGG	AAACCCGTAACATCCCTGGAGAGG
drd3_GGN18	TAGGGGGCTGTAGTTTCCCCCA	AAACTGGGGGAAACTACAGCCC
drd3_GGN20	TAGGCTGGGCTGTAGTTTCCCCCA	AAACTGGGGGAAACTACAGCCCAG
